# Impact of Social Reference Cues on Misinformation Sharing on Social Media: Series of Experimental Studies

**DOI:** 10.2196/45583

**Published:** 2023-08-24

**Authors:** Christopher M Jones, Daniel Diethei, Johannes Schöning, Rehana Shrestha, Tina Jahnel, Benjamin Schüz

**Affiliations:** 1 Institute for Public Health and Nursing Research University of Bremen Bremen Germany; 2 Leibniz ScienceCampus Digital Public Health Bremen Germany; 3 School of Computer Science University of St Gallen St Gallen Switzerland

**Keywords:** misinformation, social media, health literacy, COVID-19, fake news, Twitter, tweet, infodemiology, information behavior, information sharing, sharing behavior, behavior change, social cue, social reference, flag

## Abstract

**Background:**

Health-related misinformation on social media is a key challenge to effective and timely public health responses. Existing mitigation measures include flagging misinformation or providing links to correct information, but they have not yet targeted social processes. Current approaches focus on increasing scrutiny, providing corrections to misinformation (debunking), or alerting users prospectively about future misinformation (prebunking and inoculation). Here, we provide a test of a complementary strategy that focuses on the social processes inherent in social media use, in particular, social reinforcement, social identity, and injunctive norms.

**Objective:**

This study aimed to examine whether providing balanced social reference cues (ie, cues that provide information on users sharing and, more importantly, *not* sharing specific content) in addition to flagging COVID-19–related misinformation leads to reductions in sharing behavior and improvement in overall sharing quality.

**Methods:**

A total of 3 field experiments were conducted on Twitter’s native social media feed (via a newly developed browser extension). Participants’ feed was augmented to include misleading and control information, resulting in 4 groups: no-information control, Twitter’s own misinformation warning (misinformation flag), social cue only, and combined misinformation flag and social cue. We tracked the content shared or liked by participants. Participants were provided with social information by referencing either their *personal* network on Twitter or all Twitter users.

**Results:**

A total of 1424 Twitter users participated in 3 studies (n=824, n=322, and n=278). Across all 3 studies, we found that social cues that reference users’ personal network combined with a misinformation flag reduced the sharing of misleading but not control information and improved overall sharing quality. We show that this improvement could be driven by a change in injunctive social norms (study 2) but not social identity (study 3).

**Conclusions:**

Social reference cues combined with misinformation flags can significantly and meaningfully reduce the amount of COVID-19–related misinformation shared and improve overall sharing quality. They are a feasible and scalable way to effectively curb the sharing of COVID-19–related misinformation on social media.

## Introduction

### Background

Misleading or false health-related information on social media poses a substantial challenge for both public institutions and individuals alike [1]. During the COVID-19 pandemic, the proliferation of misinformation hindered efforts to control the spread of the virus. It has undermined the adoption of nonpharmaceutical interventions such as masking and social distancing, and it has created fear and uncertainty around vaccination [2]. In fact, >17,000 unique instances of COVID-19–related misinformation have been reported by the fact-checking collective Poynter [3]. This misinformation is often shared repeatedly, creating a multitude of misleading social media posts that spread faster and wider than accurate content [4,5]. Ironically, the architecture and algorithms of social media platforms themselves facilitate the sharing of misinformation [6], and the platforms’ own countermeasures, such as warning labels and fact-checking, have proven ineffective at a large scale [7]. Consequently, there is an urgent need to devise effective strategies to curb the dissemination of misleading content on social media platforms [1].

To date, most research efforts to combat the spread of misinformation have focused primarily on information processing. The underlying assumption is that social media users operate in an information-rich and attention-demanding environment with limited time, often lacking the cognitive resources or knowledge to assess the accuracy of the information they encounter [8]. This assumption has been supported by several key studies showing that when users gain knowledge or their attention is drawn to the accuracy of a post, they are less likely to share false information [8,9]. Unfortunately, most interventions based on these findings have proven difficult to implement and scale and, more importantly, have failed to account for the social dynamics of social media environments that promote user engagement and sharing through social cues such as “likes” and “shares” [10,11]. These social environments and cues may, in fact, have a stronger influence on users’ behavior than the actual content of the posts [12,13], especially when cognitive resources are limited or traditional credibility cues are absent [14,15].

Every piece of information shared on social media also carries social cues shaping users’ perceptions, attitudes, and behaviors [16]. This social information operates on 3 levels. First, as most social media platforms only provide very few cues to judge information credibility (eg, source expertise or trustworthiness), users have been shown to rely on engagement metrics instead when forming or maintaining beliefs and deciding what to share themselves [15,17]. Second, others endorsing content by liking or sharing provides users with normative information: the quality, frequency, and amount of behavior provide *descriptive* normative information, whereas perceptions of whether others would approve or disapprove of behavior constitute *injunctive* social norms [18,19]. Injunctive norms, in turn, affect user behavior [20]; their reporting of potentially false information [21]; and their intention to share content [22]. Third, composing and sharing messages on social media are also used to signal identity [23], that is, users may choose to express views to facilitate association with others who might share similar views and values. Social identity theory [24] describes these processes as related to in-group identification (the degree to which a person identifies with a particular social group) and intergroup bias (whether they favor this in-group over other outgroups). Sharing content on social media on particular topics can serve the function of increasing identification with a particular group and increase the difference to, for example, political outgroups [25]. Recent research on tweets by Democratic and Republican politicians on preventative measures (eg, mask wearing) supports these assumptions. Specifically, the study finds that party loyalty was associated with lesser mask promotion by Republicans, and stronger mask promotion by Democrats, respectively [26].

Despite all these implications, platforms provide social cues as very 1-sided endorsements to strengthen user engagement, resulting in an underrepresentation of dissenting views. This imbalance deprives the users of critical normative information and can influence their perceptions, decision-making, and the algorithms that determine the content displayed in users’ feeds. Targeting social processes could thus be an important additional building block to design interventions and environments that effectively reduce the sharing of misinformation on social media and empower its users, especially when cognitive and attentional resources are scarce [14] or when other common cues of information credibility are lacking [15].

### This Research

**Figure 1:**
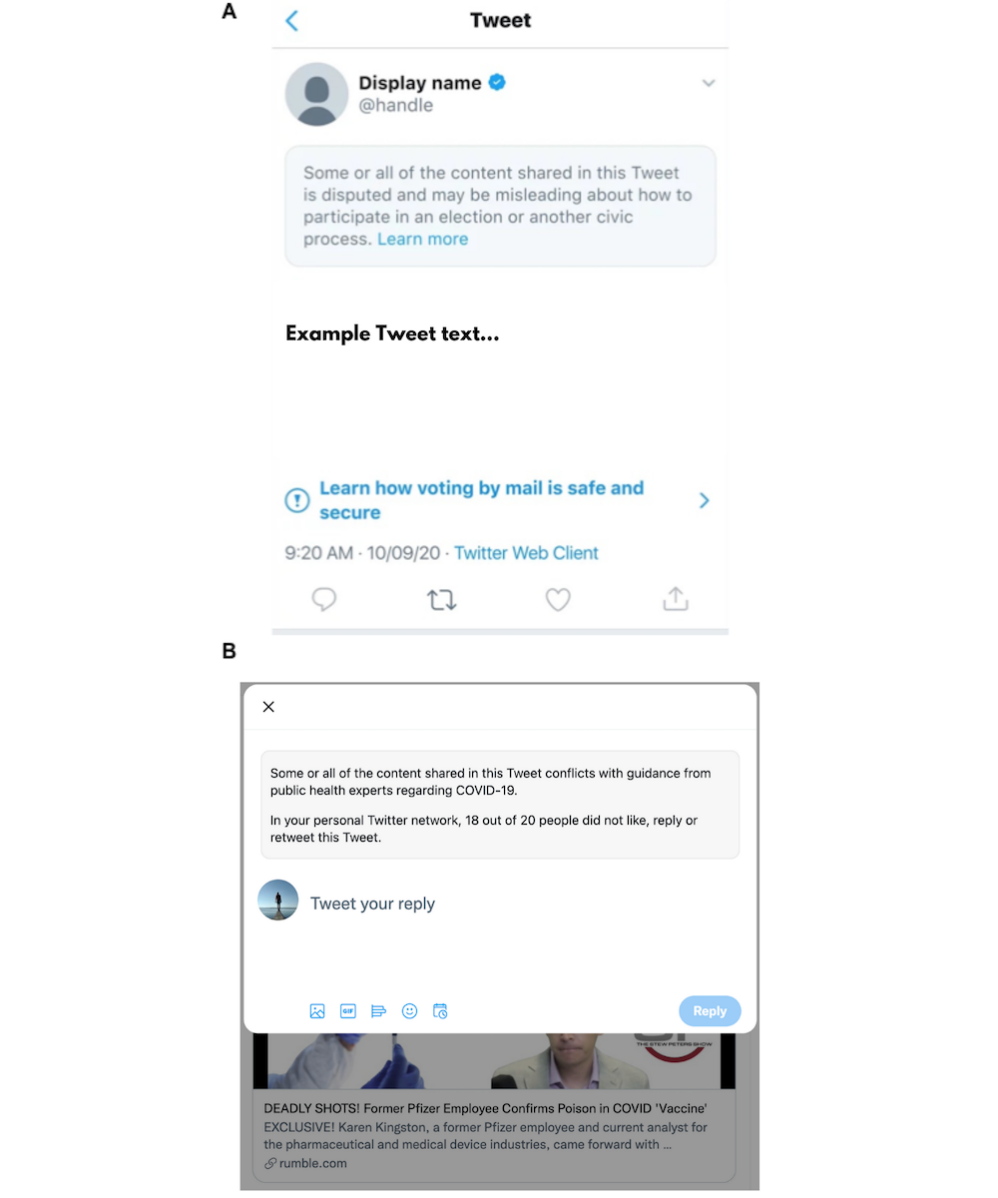
Examples of (A) Twitter’s default misinformation label in 2020 and (B) misinformation label together with our social reference cue (personal network).

In this study, we examined the effects of social cues that provide users with more balanced information on what others in their personal network and on Twitter in general share and, most importantly, do *not* share. We combined these social cues with 1 of the existing countermeasures of Twitter, namely, misinformation labels (Figure 1). In 2 subsequent studies, we examined the contribution of 2 different social reference groups, namely, the personal network of the participants and all Twitter users, and of 2 hypothesized mechanisms behind social cues, namely, changes in social norms and social identity. Using a newly developed open-source experimental paradigm to augment Twitter users’ personal feed (dan91/tweet-recommender), we provide evidence that it is possible to implement more balanced social reference information within the general user interface of social media platforms. More importantly, such social reference cues, together with the standard platform misinformation flags, can lead to reduced sharing of misinformation and improved overall sharing quality. Importantly, we experimentally examined these interventions within the natural environment of a widely used social network platform (Twitter), thereby increasing the external validity of any potential effects and supporting its potential scalability.

Specifically, we tested the following hypotheses (hypotheses 1A-2A were tested in study 1, and hypotheses 2A-3C were tested in studies 2 and 3):

Hypothesis 1A: Participants across all intervention groups share less tweets containing health-related misinformation than participants in the control group.

Hypothesis 1B: Participants across all intervention groups do not share less tweets containing health-related control information than participants in the control group.

Hypothesis 1C: Participants across all intervention groups show better *discernment* (smaller difference between misinformation and control information) than participants in the control group.

Hypothesis 2A: Participants who see a balanced social cue share less tweets containing health-related misinformation than participants in the control group.

Hypothesis 2B: Participants who see a balanced social cue do not share less tweets containing health-related control than participants in the control group.

Hypothesis 2C: Participants who see a balanced social cue show a smaller ratio of shared misinformation and control tweets than participants in the control group (indicating improved discernment).

Hypothesis 3A: Participants in the social reference cues group and those in the combined group report less approval of sharing misleading information than participants in the control group (injunctive norms).

Hypothesis 3B: Participants in the social reference cues group and those in the combined group perceive that others share less misleading information than participants in the control group (descriptive norms).

Hypothesis 3C: Participants in the social reference cues group and those in the combined group report stronger intergroup bias than participants in the control group.

## Methods

### Overview

In 3 intervention studies, we tested whether balanced social cues can (1) reduce users’ sharing of misleading content, (2) reduce their sharing of control stimuli, (3) improve their overall discernment (difference between shared misinformation and control information); (4) whether there are differences between different social reference groups (personal networks vs entirety of Twitter users); and (5) which social processes might drive these processes. As we aimed to compare our social cues intervention with Twitter’s existing countermeasure, namely, their misinformation label, but also examine potential synergetic effects, we randomly assigned participants to 1 of 4 experimental groups. Thus, participants either saw (1) only balanced social cues; (2) only Twitter’s misinformation flag; or (3) both cues and flag when liking, retweeting, or replying to a tweet. The participants in the fourth group were displayed no intervention (control). In 2 subsequent studies, we examined different social reference groups (users’ personal networks or the entirety of Twitter users) for social cues and the contribution of 2 hypothesized mechanisms behind the social cues, namely, changes in social norms and social identity. In total, we conducted 3 separate experimental studies that shared the same overall paradigm and similar procedures. We created an open-source browser extension to access and augment Twitter users’ social media feed with Twitter’s standard misinformation flags, balanced social cues, or both (Figure 1).

### Sample Size

We conducted an a priori power analysis to determine the sample size. For study 1, the sample size to be able to detect small differences in the expected rate of events (between 2 negative binomial rates; eg, μ_1_=2 and μ_2_=3) with a power of 0.90 and an α level of .05, a total sample size of n=800 was deemed sufficient (n=200 per group). For studies 2 and 3, based on simulation studies, we targeted a minimum sample size of n=260 to detect a growth condition between the last and the first measurements of Cohen *d*=0.20 with 3 repeated measurements, an α level of .05, and a power of 0.90 [27]. This minimum sample size for the growth condition exceeds the required sample size to detect medium-sized intercept and slope differences between groups with a power of 0.90 and an α level of .05 [28].

### Ethics Approval

This study was approved by the University of St. Gallen Ethics Board (HSG-EC-20210816A).

### Informed Consent and Participation

The participants provided informed consent before data collection. As we initially deceived the participants about the study aims, they were debriefed about the true aims after data collection. All data were collected using the web-based crowdsourcing platform Prolific and the German survey platform SoSci Survey. Respondents were paid a fee deemed appropriate by Prolific, equivalent to £7.50 (US $ 9.93) per hour.

### Study 1: Intervention

#### Participants

The crowdsourcing platform Prolific Academic was used to recruit participants. We invited 900 English-speaking participants with a minimum age of 18 years and >100 posts submitted on Twitter in the last 12 months. We collected all the data for study 1 on September 3, 2021. As several participants dropped out before installing the browser extension, our final sample consisted of 824 individuals aged between 18 and 65 years (mean 26.24, SD 8.59 years). Participants were either unemployed (and job seeking; 240/824, 29.1%), in full-time employment (233/824, 28.3%), or in part-time employment (121/824, 14.7%). A total of 51.3% (423/824) of the participants had at least an undergraduate degree, whereas only 1.1% (9/824) of the participants reported having no formal education. Most participants reported the following countries as their current place of residence: the United Kingdom (132/824, 16%), South Africa (123/824, 14.9%), Portugal (122/824, 14.8%), and the United States (101/824, 12.3%). We did not exclude participants who provided full consent.

#### Procedure

To avoid expectation effects, the participants were briefed to test a tweet recommendation system. After providing consent, they then answered all prestudy questionnaires and were randomly assigned to one of four conditions: (1) social reference cues, (2) standard social media (Twitter) misinformation flag, (3) combined (misinformation flag and social reference cues), and (4) control (no flag or cues).

After being redirected to Twitter, the participants were instructed to browse and interact with their personal Twitter feed as usual for 30 minutes. During this period, participants in all groups had approximately 50% of their Twitter feed replaced with misinformation randomly drawn from a pool of 40 verified misinformation tweets and 10 control tweets. All other content of the participants’ “real” feed remained unchanged. This means that participants who read more tweets overall were exposed to more misinformation and intervention content, but the proportion of misinformation and intervention content was identical across participants. If participants had been exposed to all misinformation items from the pool in the 30 minutes, the same set of items would be shown again in a randomized order. This methodology is consistent with that of previous studies [29]. After 30 minutes, the participants were redirected to Prolific and debriefed about the actual study aims, and they provided consent again. To ensure that misperceptions did not spread among participants after being exposed to misinformation (eg, attaining an antivaccination attitude), we provided them with screenshots of all tweets containing misinformation at the end of the study. In addition, a list of all false claims with corresponding corrections from the fact-checking websites was presented. When participants decided to share or like the misinformation tweets generated by our system, these interactions were intercepted to ensure that they were not visible on their Twitter profiles.

#### Material

##### Measures

We obtained demographic data (eg, age, first language, and employment status) from Prolific. Participants also completed prestudy self-report measures on education and on different potential mediators of the relationship between intervention and sharing behavior. We assessed *digital health literacy* with 4 items asking about their subjective ability to browse, search, and find as well as assess the quality and trustworthiness of digital health–related content (the codebook documenting all measures included in this study can be found in the Multimedia Appendix 1). To comprehensively assess digitally skilled participants’ abilities to find and process health-related information and reduce participant burden, we created a 4-item short scale in accordance with ability-oriented conceptions of digital literacy [30] and the European Health Literacy Survey Questionnaire 16 [31] (example item: “How confident are you evaluating the quality and trustworthiness of digital health-related content?”; for all items, see the codebook on the Open Science Framework). Participants were asked to answer each question on a 7-point scale (anchored: “not at all confident” to “very confident”). The short scale exhibited good reliability, with *ω_u_*=0.82 representing the proportion of total score variance because of a common single factor (Cronbach α=.80).

In addition, we asked participants to rate their *political orientation* on a continuum (ranging from 1 “left” to 10 “right”): “Many people use the terms ‘left’ and ‘right’ when they want to describe different political views. Thinking of your own political views, where would you place these on this scale?” The distribution of the participants’ answers was skewed toward politically more left-leaning views (mean 3.49, SD 2.33).

##### Primary Outcomes

The primary outcomes *likes* and *retweets* of misinformation and control tweets were collected through the browser extension. Note that the actual liking, sharing, or retweeting was intercepted through the browser extension.

##### Secondary Outcome

We operationalized *discernment* as the number of shared control tweets subtracted from the number of shared misinformation tweets.

##### Experimental Procedures

We developed a study-specific *Chrome browser extension* to augment participants’ actual Twitter feed. This browser extension enabled us to add manipulated tweets to participants’ natural feed (eg, content shared or posted by friends or other people users followed), record all manipulated content they viewed, and record all interactions with manipulated content. We stored all the interaction data (tweet ID, time stamp, and experimental condition) on a Google Firebase server. To protect the participants from spreading misleading information, the browser extension intercepted all likes, replies, and retweets. We will share all codes for this browser extension upon publication of this manuscript on GitHub (dan91/tweet-recommender). The extension is written in JavaScript and published on the Google Chrome Web Store but is only accessible via a direct link to prevent dissemination outside the study context.

##### Intervention Material

In total, we fed up to 50 manipulated tweets into the users’ timelines. A total of 40 of the manipulated tweets contained false claims and 10 contained short vaccine positive–, entertainment-, and sports-related information and served as *control stimuli*. *Misinformation* tweets were actual misinformation tweets collected through the Google Fact Check application programming interface. Through the application programming interface, we accessed claims that had been fact-checked by platforms such as PolitiFact or Vera Files, which are mostly not-for-profit organizations. We included tweets with the keyword “COVID” that were rated as “false.” A full list of the stimuli used can be found on the Open Science Framework. A total of 6 of these tweets cited newspapers or scientific articles.

For the *balanced social cue*, users were provided with the number of users within their own personal network and within the entirety of Twitter that saw but did not interact with the content (eg, personal network: “In your personal Twitter network, 710 out of 740 people saw but did not like, reply or retweet this tweet.” and entirety of Twitter: “On Twitter, 7400 people saw but did not like, reply or retweet this tweet.”). Importantly, we did not calculate the size of participants’ actual Twitter network. Instead, the proportion of participants’ personal Twitter network and the total number of Twitter users not reacting to the tweet were calculated based on the number of retweets of the actual misinformation content. For the personal network, the retweet count was multiplied by 10 and for the complete Twitter network by 500. The proportion of users in a person’s network who had *seen* but *ignored* a tweet was then randomized between 95% and 99%. For example, if the original misinformation tweet had been retweeted 10 times, the social reference message could read “...95 of the people in your personal network saw but did not share...On Twitter, 4950 other users saw but did not share...”

#### Analysis

The *primary outcome* variable was the number of shared misinformation tweets (sum of liked and retweeted misinformation tweets per participant). To account for the high number of participants sharing no tweets and thus a highly skewed outcome distribution (mean 1.54, SD 3.43; σ^2^=11.74), we estimated the following negative binomial regression model to test hypothesis 1A:


ln(shared-tweets) = α + β_1_ (social-reference-frame) + β_2_ (misinformation flag) + β_3_ (combined) + β_4_ (age) + β_5_ (education) + β_6_ (digital-literacy) + β_7_ (political-orientation) + ln_t_

The model was estimated using maximum likelihood estimation, and we included a dummy-coded predictor for each intervention group in the model (β_1_, β_2_, and β_3_). The control group served as reference. In addition, all models controlled for participants’ age, education, digital health literacy, and political orientation. We report regression coefficients, SEs, and incidence rate ratios as changes in Y per 1-unit increase in a predictor as effect size. The analyses were preregistered and can be retrieved from AsPredicted (preregistration ID: ju2c7). We then predicted the number of shared control tweets (the sum of liked and retweeted control tweets per participant). Again, we had to account for a highly skewed outcome distribution (mean 0.60, SD 1.45; σ^2^=2.11), and we estimated a negative binomial regression model to test hypothesis 1B. This model included the same predictors as the model described regarding hypothesis 1A. For correlational information on the predictors included in these models, please see Table S1 in Multimedia Appendix 2.

The *secondary outcome* was user discernment (operationalized as the number of shared control tweets subtracted from the number of shared misinformation tweets). Here, we estimated a linear regression model to test hypothesis 1C. This model included the same predictors as the model described regarding hypothesis 1A.

Next, we describe 2 sets of post hoc analyses in studies 2 and 3 to explore the potential mechanisms underlying the observed intervention effects.

### Studies 2 and 3: Social Reference, Social Norms, and Social Identity

#### Overview

In studies 2 and 3, we examined whether providing users with a reference to their personal network, Twitter users overall, or both differentially impacts their sharing behavior. In addition, we explored 2 potential mechanisms behind the intervention effects by examining whether repeated exposure to balanced social information induces changes in social norms (study 2) or changes in social identity (study 3) might drive the reported intervention effects. To accurately track these *changes*, we slightly changed the experimental paradigm and repeatedly assessed the target constructs after each of the several short experimental trials for each participant.

#### Participants

The crowdsourcing platform Prolific Academic was used to recruit all participants for studies 2 and 3. In total, 650 English-speaking participants were invited. We collected all data for studies 2 and 3 on November 25, 2021. As several participants had to be dropped from the final sample because they did not provide consent after being briefed about the true study aims, we reported a final sample size of n=322 for study 2 and n=278 for study 3. We did not exclude participants who provided full consent. Again, the minimum age was 18 years, and we only invited users with >100 posts submitted on Twitter in the last 12 months. Participants were aged between 18 and 76 years (mean 28.02, SD 9.77 years) and either unemployed (and job seeking; 151/600, 25.2%), in full-time employment (207/600, 34.5%), or in part-time employment (108/600, 18%).

#### Procedure

The procedure for studies 2 and 3 was similar to that of study 1. Again, participants were briefed that they were testing a tweet recommendation system, and after providing consent, they were randomly assigned to 1 of 4 experimental conditions. Each of the 3 intervention groups was shown Twitter’s misinformation flag and 1 of 3 different social cues that either referenced only the user’s personal network, all Twitter users, or combined both. Thus, the four conditions were as follows: (1) social reference cues (reference: all Twitter users) and misinformation flag, (2) social reference cues (reference: personal network) and misinformation flag, (3) combined (reference with both social reference points and misinformation flag), and (4) control (no flag or frame).

After being redirected to Chrome, participants were instructed to browse and interact with their personal Twitter feed as usual for three 5-minute trials. Again, participants in all groups had approximately 50% of their Twitter feed replaced with augmented tweets (misinformation and controls, as described for study 1). All other parameters remained unchanged. If participants had seen all pieces of augmented tweets, they would repeat in a randomized order (see study 1). After each trial, the participants were redirected to the survey platform and asked to answer questions on social norms (study 2) or intergroup bias–related traits (study 3). After 3 trials, the participants were debriefed about the true study aims, and consent was provided again. In addition, the participants in study 3 answered the postassessment measures on in-group identification. As described in the section regarding our experimental procedures, we ensured that misperceptions did not spread among participants after they were exposed to misinformation by providing them with screenshots of all misinformation tweets at the end of the study.

#### Material

*For both studies*, we again obtained demographic data (eg, age, first language, and employment status) from Prolific.

*In study 2*, we assessed subjective descriptive and injunctive norms using 1 item each after each trial. Regarding injunctive norms, we asked participants to rate the following statement: “The people I care about in my personal Twitter network approve of me sharing Covid-19-related information such as the ones I just saw” (range 1-7, anchored: “not at all” to “very strongly”). Regarding descriptive norms, the statement to be completed with the fitting assessment read: “The people I care about in my personal Twitter network...share Covid-19-related information such as the ones I just saw” (range 1-7, anchored: “[almost] never” to “[almost] always”).

*In study 3*, we assessed the intergroup bias on positive and negative traits after each trial. Intergroup bias–related traits were, for example, “intelligent,” “trustworthy,” or “gullible,” and participants rated how well those described their personal Twitter network and others with similar or different opinions on COVID-19 (range 1-7). All answers were used to compute an average score for each participant, with higher values indicating stronger bias. After completing all trials, participants answered 3 questions about their in-group identification regarding other users sharing their personal views on COVID-19 (example item: “People who have opinions on Covid-19 similar to mine have a lot in common with each other.”; range 1-7, anchored: “strongly disagree” to “strongly agree”).

#### Analysis

##### Overview

To test hypotheses 2A, 2B, and 2C, we pooled the experimental data of study 2 and study 3. We not only had to account for the high number of participants sharing no tweets, and thus highly skewed outcome distributions (2A and 2B), but also for the nonindependence of repeated assessments across experimental trials within participants. Thus, we estimated negative binomial generalized linear mixed-effects models to examine hypotheses 2A and 2B and a linear mixed-effects model to examine hypothesis 2C.

The models were estimated using maximum likelihood estimation and, in the case of the 2 negative binomial mixed-effects models, with the bound optimization by quadratic approximation optimizer. The models again included a dummy-coded predictor for each intervention group in the model (β_1_, β_2_, and β_3_), with the control group serving as reference.

To test hypotheses 3A, 3B, and 3C, we estimated 3 separate first-order linear latent growth curve models (for a graphical representation of the first model for study 2, see Figure S1 in Multimedia Appendix 2). We tested whether the intervention effects were associated with the average (ie, intercept) of participants’ injunctive norms (hypothesis 3A), descriptive norms (hypothesis 3B), intergroup bias (hypothesis 3C), or change (ie, slope) thereof. Dummy-coded predictors for each intervention group were included in the model to test the associations with the latent intercept or slope.

##### Deviation From Preregistration

We have published this manuscript as a preprint and have received valuable feedback from the research community. As a result, we now diverge from our preregistration as we had to make several key adjustments to our analytic strategy after being made aware of the several important methodological issues that could otherwise have biased our results. First, we learned that including the amount of misinformation participants had seen in the model might introduce posttreatment bias [32]. Accordingly, we dropped the amount of misinformation as a predictor from all models. Second, we were made aware that examining only the amount of shared misinformation might lead to false claims about the overall effectiveness of the intervention. Thus, we have now added new analyses to also examine the effects on users’ sharing of control stimuli as well as a change in the difference between misinformation and control information shared. Although we have now diverged from our preregistration substantially, the described changes are important to ensure the overall quality of this study. Please refer to Multimedia Appendix 2 for all other preregistered analyses that are not reported here.

In the preregistration, we had also planned to include intention to not share misleading information, their momentary self-control, and distrust of experts and intellectuals (anti-intellectualism). However, none of these variables contributed significantly as a predictor, and all other estimates also remained unchanged across both models. For the results of all other estimated models described in the preregistration, please see Tables S2 to S5 in Multimedia Appendix 2. All other divergences from the preregistration are described in the paragraph above, and we now focus on inferences based on the more parsimonious model described in the *Analysis* section regarding hypothesis 1A.

## Results

### Study 1: Intervention

We begin by presenting the results regarding the effects of social reference cues (personal network and all Twitter users) on sharing misinformation tweets, control tweets, and discernment (Table 1 depicts descriptive information on all 3 outcomes).

We found partial support for hypothesis 1A, as participants in the misinformation flag and the combined group shared less health-related misinformation than participants in the control group. However, participants in the social cue and misinformation flag groups also shared less control tweets (hypothesis 1B), whereas only participants who saw the combined social cue and misinformation flag showed an improved discernment of shared misinformation and control information (hypothesis 1C; Table 2).

**Figure 2:**
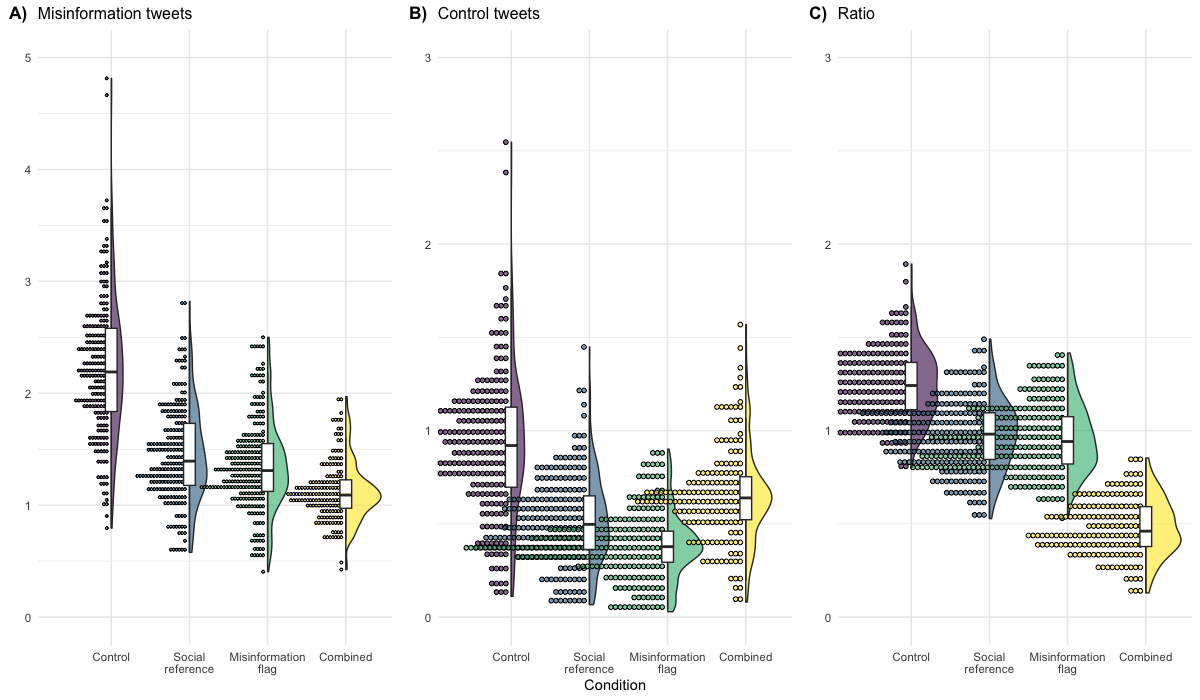
Density plots, boxplots, and stacked data points for model-based predictions of the (A) count of shared misinformation tweets, (B) count of shared control tweets, and (C) ratio of shared misinformation and control tweets per experimental condition (in study 1).

Participants in the combined intervention group shared, on average, only half of the amount of misinformation compared with the control group (b=−0.66, 95% CI −1.11 to −0.20; *t_816_*=−2.86, *P*=.004). As the thin tail of the model-based prediction distribution in Figure 2 demonstrates, presenting users with the combined intervention significantly reduced high sharing rates and thus “condensed” the distribution. This finding is especially important, as few users usually account for most of the shared information [33]. Thus, reducing their activity may have additive effects on the amount shared overall.

**Table 1:** Descriptive summary of the number of misinformation and control information pieces users shared in study 1 by experimental group: means, SDs, and highest values.

			Misinformation							Control							Discernment				
			Shared		Liked		Retweeted		Shared			Liked		Retweeted		Shared			Liked		Retweeted
**Control**																					
	Mean (SD)	2.16 (3.91)		2.16 (3.83)		0.27 (0.77)		0.92 (1.77)			0.95 (1.85)		0.05 (0.25)		1.23 (3.41)			1.21 (3.36)		0.22 (0.72)	
	Maximum	26.00		25.00		4.00		12.00			13.00		2.00		19.00			19.00		4.00	
**Misinformation flag**																					
	Mean (SD)	1.37 (3.34)		1.27 (3.12)		0.17 (0.73)		0.40 (1.21)			0.40 (1.21)		0.03 (0.18)		0.97 (2.74)			0.87 (2.56)		0.14 (0.72)	
	Maximum	26.00		26.00		6.00		10.00			10.00		2.00		19.00			19.00		6.00	
**Social cue**																					
	Mean (SD)	1.46 (3.63)		1.28 (3.06)		0.28 (1.26)		0.49 (1.39)			0.45 (1.22)		0.08 (0.39)		0.97 (2.67)			0.83 (2.38)		0.21 (0.94)	
	Maximum	23.00		22.00		14.00		9.00			8.00		4.00		18.00			15.00		10.00	
**Combined**																					
	Mean (SD)	1.12 (2.29)		1.12 (2.17)		0.09 (0.40)		0.63 (1.34)			0.60 (1.29)		0.04 (0.26)		0.49 (1.84)			0.52 (1.79)		0.05 (0.37)	
	Maximum	15.00		12.00		3.00		7.00			7.00		2.00		10.00			10.00		2.00	

**Table 2:** Summary of negative binomial regression model predicting count of shared misinformation tweets^a^, negative binomial regression model predicting count of shared control tweets, and linear regression model predicting ratio of shared misinformation and control tweets in study 1.

	Misinformation				Control				Discernment	
	Estimate (SE)	IRR^b^	*P* value	Estimate (SE)		IRR	*P* value	Estimate (SE)		*P* value
Intercept	1.60 (0.56)	4.97	*.004* ^c^	1.20 (0.62)		3.32	.05	1.91 (0.72)		*.008*
Group: social cue	−0.40 (0.21)	0.67	.06	−0.54 (0.23)		0.58	*.01*	−0.26 (0.27)		.33
Group: misinformation flag	−0.43 (0.21)	0.65	*.03*	−0.78 (0.23)		0.46	*.001*	−0.25 (0.27)		.35
Group: combined	−0.66 (0.23)	0.52	*.004*	−0.32 (0.24)		0.72	.18	−0.75 (0.29)		*.01*
Age	−0.02 (0.01)	0.98	*.01*	−0.06 (0.01)		0.94	*<.001*	−0.01 (0.01)		.63
Education	−0.02 (0.05)	0.98	.70	0.00 (0.06)		1.00	.99	−0.03 (0.07)		.70
Digital literacy	−0.08 (0.08)	0.93	.36	−0.05 (0.09)		0.95	.55	−0.10 (0.11)		.34
Political orientation	0.09 (0.03)	1.09	*.009*	0.11 (0.04)		1.11	*.003*	0.06 (0.04)		.15

^a^Estimated model 1: θ=0.25; and model 2: θ=0.26.

^b^IRR: incidence rate ratio (percentage change in the dependent variable per 1-unit change in the predictor, either >1 or <1).

^c^*P* values are italicized if significant at *P*<.05.

Beyond the intervention group, age and self-reported political orientation were significantly associated with the amount of misinformation shared. A 1-point stronger right-wing political orientation was associated with an average 9% increase in sharing (b=0.09, 95% CI 0.02-0.16; *t_816_*=2.62, *P*=.009), whereas 1 additional year of age was associated with an average 2% decrease in sharing (b=−0.02, 95% CI −0.04 to 0.00; *t_816_*=−2.46, *P*=.01).

### Studies 2 and 3: Social Reference, Social Norms, and Social Identity

Across studies 2 and 3, we examined whether the social reference users were provided with was important for the overall effect.

**Figure 3:**
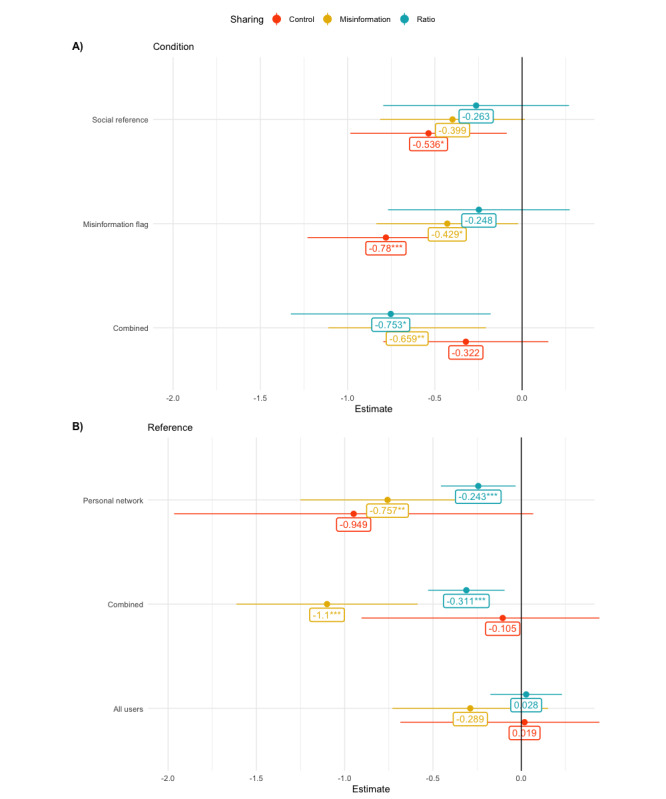
Studies 1 to 3: effect plot depicting model estimates across models for shared misinformation tweets, shared control tweets and ratio thereof. (A) Depicts the main effects of experimental conditions on all outcomes in study 1 and (B) depicts the main effects of different social references combined with misinformation flag in studies 2 and 3. * denotes *P*<.05, ** denotes *P*<.01, *** denotes *P*<.001.

We found that participants shared less misinformation and improved their overall sharing quality (discernment) when provided with a cue referencing their personal network or their personal network and all Twitter users (hypothesis 2A and hypothesis 2C; Table 3). The amount of shared control information was not significantly lower than that of the control group (please note the substantial CIs around some estimates; Figure 3).

In additional post hoc analyses, we found that the observed effects may have resulted from changes in injunctive social norms but not social identity (operationalized as intergroup bias). We found that participants in the personal network group reported a significantly negative slope of injunctive norms across the experimental trials, suggesting that their perceived approval of sharing misinformation declined. In contrast, this was not observed in any of the other experimental groups. This indicates that changes in social norms might contribute to the effects of social cues on participants’ sharing behaviors (hypothesis 3A; Tables 4 and 5). In contrast, we found no support for the other hypothesized mechanism, as participants in the social cues groups did not report changes to trait-related intergroup bias (hypothesis 3C).

**Table 3:** Summary of hierarchical negative binomial regression model predicting count of shared misinformation tweets, negative binomial regression model predicting count of shared control tweets, and linear regression model predicting discernment with pooled data of studies 2 and 3.

	Misinformation			Control			Discernment	
	Estimate (SE)	*P* value	Estimate (SE)		*P* value	Estimate (SE)		*P* value
Intercept	−1.70 (0.26)	*<.001* ^a^	−5.74 (0.62)		*<.001*	0.55 (0.11)		*<.001*
Trial	0.00 (0.08)	>.99	0.68 (0.17)		*<.001*	0.00 (0.04)		.96
Reference: personal network	−0.76 (0.25)	*.003*	−0.95 (0.52)		.06	−0.24 (0.11)		*.02*
Reference: combined	−1.10 (0.26)	*<.001*	−0.11 (0.41)		.79	−0.31 (0.11)		*.005*
Reference: all users	−0.29 (0.22)	.19	0.02 (0.36)		.95	0.03 (0.10)		.78

^a^Predictors are italicized if significant at *P*<.05.

**Table 4:** Summary of regression estimates of the effects of different social cue references on intercepts and slopes across latent growth curve models predicting injunctive norms, descriptive norms, and intergroup bias across experimental trials.

		Model 1			Model 2			Model 3	
		Estimate (SE)	*P* value	Estimate (SE)		*P* value	Estimate (SE)		*P* value
**Intercept combined**		0.157 (0.332)	.63	0.218 (0.317)		.49	−0.1 (0.198)		.61
	Personal network	0.149 (0.335)	.65	−0.006 (0.32)		.98	−0.169 (0.189)		.37
	All users	0.311 (0.336)	.35	0.322 (0.321)		.31	−0.062 (0.182)		.73
**Slope combined**		−0.158 (0.117)	.17	−0.019 (0.108)		.85	0.033 (0.108)		.75
	Personal network	−0.294 (0.119)	*.01* ^a^	−0.129 (0.109)		.23	−0.107 (0.103)		.29
	All users	−0.178 (0.119)	.13	−0.171 (0.11)		.11	−0.012 (0.099)		.90

^a^Predictors are italicized if significant at *P*<.05.

**Table 5:** Fit indices for the 3 latent growth curve models predicting injunctive norms, descriptive norms, and intergroup bias across experimental trials.

Fit measure	Estimate		
	Model 1	Model 2	Model 3
Chi-square (*df*)	11.451 (4)	10.422	11.97 (4)
*P* value for specified model	.02	.03	.02
Comparative Fit Index	0.989	0.991	0.978
Root mean square error of approximation	0.08	0.075	0.089
Standardized root mean square residual	0.019	0.016	0.026
Akaike information criterion	3096.519	2925.106	2084.848

## Discussion

### Principal Findings

The COVID-19 pandemic has highlighted the importance of understanding and potentially modifying the determinants of sharing behavior on social media. The vast amount of misleading information shared on social media has contributed to lower vaccine uptake, increased partisan attitudes toward public health recommendations, and mistrust in science- and evidence-based public health [2,34-36]. Together, our studies suggest that if social media users are provided with balanced social reference cues, that is, information on the sharing and *not* sharing of other individuals within their personal network, in addition to a misinformation flag, their perceived injunctive norms about the appropriateness of sharing behavior and their actual sharing behavior change. Importantly, the overall improvements in the quality of information sharing were observed only if the social information referenced users’ personal network and was combined with the misinformation flags at least previously provided by Twitter. On its own, the social reference or misinformation flag had no consistent positive effects on sharing quality. Recent research on sharing behavior has highlighted the gap between which content users rate as trustworthy and which they actually share [8], as well as the importance of “attention economy” and a focus on the accuracy of message content to modify sharing behavior [7]. Here, we provide the first evidence that in addition to a focus on message content and increasing accuracy in judging the veracity of web-based information, it is crucial to address the social determinants of sharing behavior. We argue that it is thus critical to understand information-sharing, and misinformation-sharing specifically, as a behavior determined by social processes and the specific contexts users are embedded in that have been created to foster user engagement through different social processes. Although interaction behavior within the social media environment in itself is mainly based on endorsing social cues (such as recommender systems) and social reinforcement processes (such as likes), these processes have not yet been systematically addressed in interventions to modify misinformation-sharing behavior.

Our findings in studies 2 and 3 show that the social reference cues in combination with the social network misinformation flags change users’ subjective *injunctive* norms (perception of whether behaviors are deemed acceptable and appropriate within a given context), whereas *descriptive* norms, perceptions of the frequency of a specific behavior within a social reference group, did not. This suggests that our social reference cue can provide a reference point on which behaviors are socially acceptable within users’ personal networks. Previous research suggests that the approval of important others in personal networks is of key importance for processing misinformation [37]. The social reference cue did not affect social identity in terms of in-group identification and intergroup bias, suggesting that these changes in norms were not because of a changing affiliation or identification with the users’ social networks or that our items (newly developed for this study) were not able to capture changes in social identity processes. Together, these findings highlight that normative perceptions play an important role in decision-making under load, that is, in conditions with limited time, large amounts of information to be judged, and other common cues of information credibility lacking [15]. Interestingly, recent research has also found a combination of refutation and graphically presented social norms to have the greatest impact on belief change [38]. The fact that our study replicates the effects found in earlier studies increases our confidence in interpreting the intervention effects as generalizable. Fittingly, we also found between-participant differences in sharing behavior, such that there were few users who shared substantial amounts of misinformation (up to 26 tweets in 30 min), whereas many others did not engage in sharing behavior at all [33,39]. Similarly, we found that the general political orientation of participants influenced their sharing behavior, such that, in particular, participants endorsing right-wing and authoritarian statements were more likely to share COVID-19–related misinformation [39,40]. This also points to the potential of exploring whether our findings replicate beyond the context of COVID-19–related misinformation.

### Limitations and Strengths

Our findings are to be considered in the light of some substantial limitations. First, as our paradigm had no access to and thus could not analyze the actual amount of misinformation shared within participants’ personal networks, the social reference cues (ie, the percentages reported) were a priori set by us. It remains to be seen whether basing the reference cues on actual shares has similar effects. It is especially important to test how lower rates of nonshares affect users’ behavior. Considering the well-documented negative effects of so-called *echo chambers*, adding social cues with low frequencies of not sharing or liking tweets (eg, “In your personal network, 7 out of 100 users did not like, reply, or retweet this Tweet”) might actually reinforce negative sharing patterns and the overall spread within these networks. Examining such potential negative effects is crucial before evaluating the overall effectiveness of social approaches in intervention development. Second, the significant results in study 2 fall just under the arbitrary α level of .05. Thus, we only interpreted them as the first indication of a potential mechanism and not sufficient evidence to rest major claims on. Third, the time frame within our paradigm was very short in relation to the usually vast amount of information read before sharing and a usually lower percentage of fake news in users’ feeds [33]. Thus, we added a very high amount of misinformation content into participants’ feeds, and it remains to be tested whether the intervention effects remain substantial over longer time frames and with a (more realistic) lower density of misinformation. Fourth, as we did not assess users’ beliefs in the misleading pieces of information (and control pieces), we cannot examine whether the intervention introduced, for example, an overly critical assessment across all information. Thus, we cannot examine the overall effectiveness of the intervention—something future research could address by using this study’s paradigm to assess user beliefs.

In contrast, our experimental paradigm has good external validity; by embedding our social reference cues within the general platform of a social media network, we used the actual user interface that the general population is exposed to. This allowed us to generalize our observed effects beyond those generated in laboratory settings [41]. At the same time, our paradigm allowed us to retain control over what participants were exposed to, thus providing participants with many different sources of existing, and thus “real,” misinformation—past research has only been able to analyze shared information from a given set of specific news sites or influential public persons. In addition, and crucially, we were able to not only add misinformation but also control information and could thus extend our analyses to overall sharing quality. In particular, in light of several studies examining only sharing rates (or rather intentions thereof) without comparing them with all other sharing activities and a ratio thereof as an indicator of discernment, our paradigm and results add important points to the discourse.

### Conclusions

Our results suggest that it is possible to implement balanced social reference cues within the general user interface of social media platforms and that these social reference cues, together with the standard platform misinformation flags, can lead to reduced sharing of web-based misinformation and improved quality of overall sharing. In the context of major challenges to public health and public trust caused by excessive and strategically placed misinformation on social media, such building blocks to effective mitigation measures have the potential to reduce the sharing of misinformation with all its associated negative consequences.

## Appendix

Multimedia Appendix 1 Codebook.

Multimedia Appendix 2 Social reference cues can reduce misinformation sharing on social media: an experimental study on Twitter.

## Data Availability

The data sets generated and analyzed during this study are available in an anonymized form on the Open Science Framework [50].

The analytic code and this reproducible manuscript are available on the Open Science Framework [50].
