# Supplementary material for: Impact of Social Reference Cues on Misinformation Sharing on Social Media: Series of Experimental Studies
Source: J Med Internet Res. 2023 Aug 24;25:e45583. doi: 10.2196/45583 (PMC10485706; doi:10.2196/45583)
Supplement: Multimedia Appendix 1 [file jmir_v25i1e45583_app1.pdf]

Study 1

topic / scale:	question:	variable name:	answers:	values:	description:	scoring:	
education	Please select the highest level of qualification you have obtained.	education	No formal qualifications;<br>Secondary school/GCSE;<br>College/A levels;<br>Undergraducate degree (BA/BSc/other);<br>Graduate degree (MA/MSc/MPhil/other);<br>Post-graduate (PhD/MD/other)	0 to 5			
digital literacy	How confident are you ...					mean across all 4 items	
	... browsing, searching and finding digital health-related content?	digital_literacy_01	not at all confident - very confident	1 to 7			
	... evaluating the quality and trustworthiness of digital health-related content?	digital_literacy_02	not at all confident - very confident	1 to 7			
	... browsing, searching and finding digital content in general?	digital_literacy_03	not at all confident - very confident	1 to 7			
	... evaluating the quality and trustworthiness of digital content in general?	digital_literacy_04	not at all confident - very confident	1 to 7			
political orientation	Many people use the terms "left" and "right" when they want to describe different political views. Thinking of your own political views, where would you place these on this scale?	political_orientation	left - right	1 to 10			

Study 2

	topic / scale:	question:	variable name:	answers:	values:	description:	scoring:
Pre	education	Please select the highest level of qualification you have obtained.	education	No formal qualifications;<br>Secondary school/GCSE;<br>College/A levels;<br>Undergraducate degree (BA/BSc/other);<br>Graduate degree (MA/MSc/MPhil/other);<br>Post-graduate (PhD/MD/other)	0 to 5		
Post (assessment block)	Injunctive norms	The people I care about in my personal Twitter network ... approve of me sharing Covid-19-related information such as the ones I just saw.	norms_injuctive	Anchored: not all - very strongly	1 to 7		
	Descriptive norms	The people I care about in my personal Twitter network ... share Covid-19-related information such as the ones I just saw.	norms_descriptive	Anchored: (almost) never - (almost) always	1 to 7		

Study 3

	topic / scale:	question:	variable name:	answers:	values:	description:	scoring:
Pre	education	Please select the highest level of qualification you have obtained.	education	No formal qualifications;<br>Secondary school/GCSE;<br>College/A levels;<br>Undergraducate degree (BA/BSc/other);<br>Graduate degree (MA/MSc/MPhil/other);<br>Post-graduate (PhD/MD/other)	0 to 5		
Post (assessment block)	Intergroup Bias on Positive and Negative Traits	**How well do the following words describe your personal Twitter network?**					
		Intelligent	ib_persnet_intelligent	does not describe at all – describes very well	1 - 7		
		Trustworthy	ib_persnet_trustworthy	does not describe at all – describes very well	1 - 7		
		Independent-minded	ib_persnet_independendminded	does not describe at all – describes very well	1 - 7		
		Aggressive	ib_persnet_aggressive	does not describe at all – describes very well	1 - 7		
		Gullible	ib_persnet_gullible	does not describe at all – describes very well	1 - 7		
		Careless	ib_persnet_careless	does not describe at all – describes very well	1 - 7		
		**How well do the following words describe others who have opinions on COVID-19 similar to you?**					
		Intelligent	ib_simopinions_intelligent	does not describe at all – describes very well	1 - 7		
		Trustworthy	ib_simopinions_trustworthy	does not describe at all – describes very well	1 - 7		
		Independent-minded	ib_simopinions_independendminded	does not describe at all – describes very well	1 - 7		
		Aggressive	ib_simopinions_aggressive	does not describe at all – describes very well	1 - 7		
		Gullible	ib_simopinions_gullible	does not describe at all – describes very well	1 - 7		
		Careless	ib_simopinions_careless	does not describe at all – describes very well	1 - 7		
		**How well do the following words describe others who do NOT have opinions on COVID-19 similar to you?**					
		Intelligent	ib_diffopinions_intelligent	does not describe at all – describes very well	1 - 7		
		Trustworthy	ib_diffopinions_trustworthy	does not describe at all – describes very well	1 - 7		
		Independent-minded	ib_diffopinions_independendminded	does not describe at all – describes very well	1 - 7		
		Aggressive	ib_diffopinions_aggressive	does not describe at all – describes very well	1 - 7		
		Gullible	ib_diffopinions_gullible	does not describe at all – describes very well	1 - 7		
		Careless	ib_diffopinions_careless	does not describe at all – describes very well	1 - 7		
Post	In-Group Identification	I feel a bond with others who have opinions on Covid-19 similar to mine.	ingroup_ident_bond	strongly disagree - strongly agree	1 - 7		
		I am similar to others who have opinions on Covid-19 similar to mine.	ingroup_ident_group_common	strongly disagree - strongly agree	1 - 7		
		People who have opinions on Covid-19 similar to mine have a lot in common with each other. [1]	ingroup_ident_similar	strongly disagree - strongly agree	1 - 7		
