# Supplementary material for: Impact of Social Reference Cues on Misinformation Sharing on Social Media: Series of Experimental Studies
Source: J Med Internet Res. 2023 Aug 24;25:e45583. doi: 10.2196/45583 (PMC10485706; doi:10.2196/45583)
Supplement: Multimedia Appendix 2 [file jmir_v25i1e45583_app2.docx]

Supplementary Information

**Social reference cues can reduce misinformation sharing on social media: An experimental study on Twitter**

Christopher M. Jones *et al.*

Corresponding author. Email: jones@uni-bremen.de

Table S1

*Correlation matrix, means and standard deviations for all predictors included in count regression models.*

| Predictor | 1 | 2 | 3 | *M* | *SD* |
| --- | --- | --- | --- | --- | --- |
| 1. Age | - |  |  | 26.24 | 8.59 |
| 2. Education | .23*** | - |  | 6.23 | 1.51 |
| 3. Digital health literacy | .15*** | .11** | - | 5.61 | 0.92 |
| 4. Political orientation | .07* | .04 | .06 | 3.49 | 2.33 |

*Note. M* and *SD* are used to represent means and standard deviations, respectively. * indicates p < .05; ** p < .01; *** p < .001.

Table S2

*Summary of negative binomial regression model predicting count of liked misinformation tweets in study 1: unstandardized regression coefficient, standard errors (SE), Wald confidence intervals, p values; IRR = incidence rate ratio.*

Wald CI

|  | *Estimate* | *SE* | *IRR* | *LL* | *UL* | *p* |
| --- | --- | --- | --- | --- | --- | --- |
| **Group: Social cue** | -0.51 | 0.21 | 0.60 | -0.91 | -0.11 | .013 |
| **Group: Misinfo flag** | -0.51 | 0.20 | 0.60 | -0.90 | -0.11 | .012 |
| **Group: Combined** | -0.65 | 0.22 | 0.52 | -1.09 | -0.21 | .004 |
| **Age** | -0.03 | 0.01 | 0.97 | -0.05 | -0.01 | .001 |
| Education | -0.01 | 0.05 | 0.99 | -0.11 | 0.09 | .785 |
| Digital health literacy | -0.08 | 0.08 | 0.92 | -0.24 | 0.08 | .325 |
| **Political orientation** | 0.08 | 0.03 | 1.08 | 0.01 | 0.14 | .018 |

*Note.* Predictors are bold if significant at p < .05; IRR represents the (percentage) change in the dependent variable per one-unit change in the predictor, either above or below 1; estimated *Theta* = 0.23.

Table S3

*Summary of negative binomial regression model predicting count of retweeted misinformation tweets in study 1: unstandardized regression coefficient, standard errors (SE), Wald confidence intervals, p values; IRR = incidence rate ratio.*

Wald CI

|  | *Estimate* | *SE* | *IRR* | *LL* | *UL* | *p* |
| --- | --- | --- | --- | --- | --- | --- |
| Group: Social cue | -0.08 | 0.38 | 0.92 | -0.82 | 0.66 | .825 |
| Group: Misinfo flag | -0.55 | 0.38 | 0.58 | -1.30 | 0.20 | .150 |
| **Group: Combined** | -1.29 | 0.47 | 0.28 | -2.21 | -0.36 | .007 |
| Age | 0.02 | 0.02 | 1.02 | -0.01 | 0.05 | .280 |
| Education | -0.03 | 0.10 | 0.97 | -0.23 | 0.16 | .722 |
| Digital health literacy | 0.05 | 0.16 | 1.06 | -0.25 | 0.36 | .732 |
| **Political orientation** | 0.18 | 0.06 | 1.20 | 0.06 | 0.30 | .002 |

*Note.* Predictors are bold if significant at p < .05; IRR represents the (percentage) change in the dependent variable per one-unit change in the predictor, either above or below 1; estimated *Theta* = 0.10.

Table S4

*Summary of negative binomial regression model predicting count of inhibited shares of misinformation tweets in study 1: unstandardized regression coefficient, standard errors (SE), Wald confidence intervals, p values; IRR = incidence rate ratio.*

Wald CI

|  | *Estimate* | *SE* | *IRR* | *LL* | *UL* | *p* |
| --- | --- | --- | --- | --- | --- | --- |
| **Group: Social cue** | 1.26 | 0.27 | 3.52 | 0.73 | 1.79 | < .001 |
| **Group: Misinfo flag** | 1.18 | 0.26 | 3.25 | 0.66 | 1.70 | < .001 |
| **Group: Combined** | 1.32 | 0.29 | 3.74 | 0.76 | 1.88 | < .001 |
| Age | -0.01 | 0.01 | 0.99 | -0.03 | 0.01 | .334 |
| Education | 0.10 | 0.06 | 1.10 | -0.03 | 0.22 | .129 |
| Digital health literacy | 0.05 | 0.10 | 1.06 | -0.15 | 0.26 | .602 |
| **Political orientation** | 0.11 | 0.04 | 1.12 | 0.04 | 0.19 | .004 |

*Note.* Predictors are bold if significant at p < .05; IRR represents the (percentage) change in the dependent variable per one-unit change in the predictor, either above or below 1; estimated *Theta* = 0.22.

Table S5

*Summary of negative binomial regression model predicting count of shared misinformation tweets in study 1 including all predictors mentioned in pre-registration: unstandardized regression coefficient, standard errors (SE), Wald confidence intervals, p values; IRR = incidence rate ratio.*

Wald CI

|  | *Estimate* | *SE* | *IRR* | *LL* | *UL* | *p* |
| --- | --- | --- | --- | --- | --- | --- |
| Group: Social cue | -0.40 | 0.21 | 0.67 | -0.82 | 0.01 | .056 |
| **Group: Misinfo flag** | -0.42 | 0.21 | 0.66 | -0.82 | -0.01 | .044 |
| **Group: Combined** | -0.63 | 0.23 | 0.53 | -1.08 | -0.18 | .006 |
| **Age** | -0.03 | 0.01 | 0.97 | -0.05 | -0.01 | .009 |
| Education | -0.02 | 0.05 | 0.98 | -0.12 | 0.08 | .706 |
| Digital health literacy | -0.09 | 0.09 | 0.92 | -0.25 | 0.08 | .309 |
| **Political orientation** | 0.08 | 0.03 | 1.08 | 0.02 | 0.14 | .016 |
| Anti-intellectualism | -0.03 | 0.08 | 0.97 | -0.20 | 0.13 | .703 |
| Intention to not share | -0.08 | 0.06 | 0.92 | -0.19 | 0.03 | .162 |
| Self-Control | 0.04 | 0.06 | 1.04 | -0.08 | 0.17 | .495 |

*Note.* Predictors are bold if significant at p < .05; IRR represents the (percentage) change in the dependent variable per one-unit change in the predictor, either above or below 1; estimated *Theta* = 0.22.

IN1

IN2

IN3

SR1

SR2

SRC

I

S

*Figure S1*. Graphical representation of latent growth curve model for change in participants’ injunctive norms across experimental trials. *SR1* depicts personal network reference, *SR2* depicts all Twitter users reference, *SRC* depicts combined reference, *IN1* - *IN3* depict the three assessments of participants’ injunctive norms. *I* and *S* depict latent intercept and slope, respectively. *SR1*, *SR2*, *SR3* are regressed on *I* and *S*.
